# Strengthening Community Belonging: Survey Evidence from a Co-Generational Pilot Program

**DOI:** 10.1093/geroni/igaf122.3846

**Published:** 2025-12-31

**Authors:** Sofia Holmes, BoRin Kim, Kelley Petralia, Laura Cerniglia, Tracy LaCroix

**Affiliations:** University of New Hampshire, Durham, New Hampshire, United States; University of New Hampshire, University of New Hampshire, New Hampshire, United States; Westborough Connects, Westborough, Massachusetts, United States; Westborough Connects, Westborough, Massachusetts, United States; Westborough Connects, Westborough, Massachusetts, United States

## Abstract

Intergenerational programs play a vital role in building inclusive communities by reducing isolation, challenging ageism, and fostering belonging across generations. Developed in collaboration with a local community organization, this study contributes to the limited quantitative evidence on their impacts. We examined pre- and post-program changes in ageism, perceived social cohesion, and social connection among participants in a community-based co-generational program. Eleven dyads of older adults (65+) and adolescents (14–17) completed structured activities weekly over six weeks, with pre- and post-program surveys administered. Paired-sample t-tests assessed within-group changes, and independent t-tests examined generational differences. The adolescent group (n = 11) was 73% female, 64% nonwhite, and over half had lived locally their entire lives. The older adult group (n = 11) was 64% female, all white, mean age 70.5; two-thirds had recently moved to town, while one-third had lived there 25+ years, and over half were single. Perceived social cohesion improved after the program (t(21)=1.743, p = 0.096), with a stronger effect among older adults. Pre-program, adolescents scored higher on the ageism scale than older adults (t(20)=-1.863, p=.077), but this gap narrowed and was no longer significant post-program (t(20)=0.746, p=.464). No significant changes were observed in social connection, though small positive trends emerged. Findings suggest that co-generational programs can enhance social cohesion and help reduce intergenerational biases. While limited by small sample size, this community–academic partnership provides preliminary evidence that can inform the development of future intergenerational initiatives, with particular potential to benefit vulnerable older adults at high risk of isolation.

